# Comparing spatial patterns of 11 common cancers in Mainland China

**DOI:** 10.1186/s12889-022-13926-y

**Published:** 2022-08-15

**Authors:** Lin Zhang, Xia Wan, Runhe Shi, Peng Gong, Yali Si

**Affiliations:** 1 Department of Earth System Science, Ministry of Education Key Laboratory for Earth System Modeling, Institute for Global Change Studies, Tsinghua University, Beijing, 100084 China; 2 Institute of Basic Medical Sciences, Chinese Academy of Medical Sciences and School of Basic Medicine, Peking Union Medical College, Beijing, 100005 China; 3 Key Laboratory of Geographic Information Science, Ministry of Education, East China Normal University, Shanghai, 200241 China; 4 Department of Geography and Department of Earth Sciences, University of Hongkong, Hongkong, 999077 China; 5 Institute of Environmental Sciences CML, Leiden University, Leiden, 2333 CC The Netherlands

**Keywords:** Cancer burden, Spatial pattern, Spatial clustering, Hotspots, Spatial analysis

## Abstract

**Background:**

A stronger spatial clustering of cancer burden indicates stronger environmental and human behavioral effects. However, which common cancers in China have stronger spatial clustering and knowledge gaps regarding the environmental and human behavioral effects have yet to be investigated. This study aimed to compare the spatial clustering degree and hotspot patterns of 11 common cancers in mainland China and discuss the potential environmental and behavioral risks underlying the patterns.

**Methods:**

Cancer incidence data recorded at 339 registries in 2014 was obtained from the “China Cancer Registry Annual Report 2017”. We calculated the spatial clustering degree of the common cancers using the global Moran’s Index and identified the hotspot patterns using the hotspot analysis.

**Results:**

We found that esophagus, stomach and liver cancer have a significantly higher spatial clustering degree ($p<0.05$) than others. When by sex, female esophagus, male stomach, male esophagus, male liver and female lung cancer had significantly higher spatial clustering degree ($p<0.001$). The spatial clustering degree of male liver was significantly higher than that of female liver cancer ($p<0.001$), whereas the spatial clustering degree of female lung was significantly higher than that of male lung cancer ($p<0.001$). The high-risk areas of esophagus and stomach cancer were mainly in North China, Huai River Basin, Yangtze River Delta and Shaanxi Province. The hotspots for liver and male liver cancer were mainly in Southeast China and south Hunan. Hotspots of female lung cancer were mainly located in the Pearl River Delta, Shandong, North and Northeast China. The Yangtze River Delta and the Pearl River Delta were high-risk areas for multiple cancers.

**Conclusions:**

The top highly clustered cancer types in mainland China included esophagus, stomach and liver cancer and, by sex, female esophagus, male stomach, male esophagus, male liver and female lung cancer. Among them, knowledge of their spatial patterns and environmental and behavioral risk factors is generally limited. Potential factors such as unhealthy diets, water pollution and climate factors have been suggested, and further investigation and validation are urgently needed, particularly for male liver cancer. This study identified the knowledge gap in understanding the spatial pattern of cancer burdens in China and offered insights into targeted cancer monitoring and control.

**Supplementary Information:**

The online version contains supplementary material available at 10.1186/s12889-022-13926-y.

## Background

Cancer is the leading cause of death in most countries and is one of the main causes of death in China [1, 2]. There were approximately 4,064,000 new cancer cases and 2,413,500 cancer deaths in China in 2016 [3]. Lung (covering the trachea, bronchus, and lung), stomach, colorectum (covering the colon, rectum, and anus), liver, breast and esophagus cancer are the most common cancers in China, accounting for more than half of the new cases [4]. In addition, the incidence rates of prostate and female thyroid cancer have also increased rapidly over the last two decades.

Cancers are chronic multifactorial diseases [5, 6]. Age, genetics, reproduction, human behavioral (e.g., diet and lifestyle) and environmental (e.g., pollution, radiation, and socioeconomic status) factors all affect cancer pathogenesis [7–10]. The impact of environmental factors on cancer, especially exposure to pollution, is gaining more attention. For example, lung cancer is correlated with particulate matter (PM2.5, PM10) air pollution [11], colorectal cancer is related to nitrate pollution in drinking water [12], and female breast cancer increases with higher light pollution at night [13]. Unhealthy diet and lifestyle, for example smoking, alcohol drinking, high intake of fat and low intake of vegetables, are common risks for cancers [14–16].

**Fig. 1:**
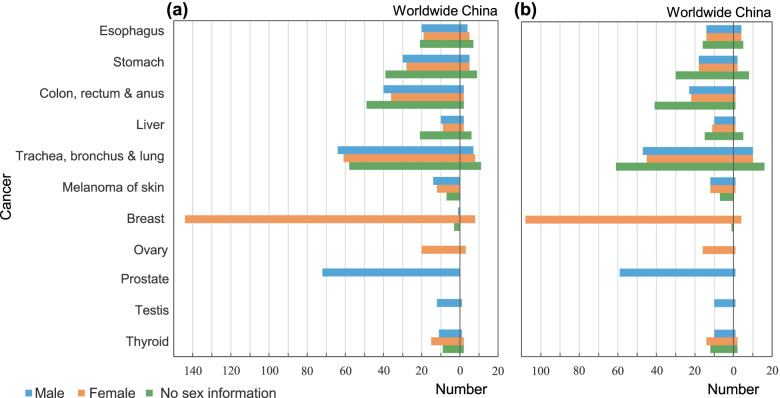
Number of studies investigating (**a**) the spatial patterns and (**b**) the environmental and human behavioral risk factors for cancers worldwide and in China from 1991 to 2021, according to Web of Science and PubMed. Key words used are ‘cancer or tumor or neoplasm’ and ‘spatial cluster* or spatial analysis or spatial pattern*’. An asterisk indicates that all terms that begin with a word followed by an asterisk will be searched. The total number of papers is 631, and only those with quantitative analyses are included

The spatial pattern of a cancer reflects the geographic distribution characteristics of its risk factors [17]. It can offer important insights into forming etiological hypotheses. Spatial clustering analysis can help detect areas with exceptionally high concentration of disease and offer insights into potential environmental and behavioral risks [18–20]. According to Web of Science and PubMed from 1991–2021, female breast, male prostate and lung cancer have attracted relatively higher attention regarding their spatial patterns and environmental and behavioral risk factors globally, while studies on melanoma of skin (hereinafter abbreviated to skin), thyroid, and liver cancer are relatively limited (Fig. 1). In China, relevant studies are generally limited, especially for thyroid, colorectum, and liver cancer (Fig. 1).

The degree of spatial clustering can reflect how much a cancer is potentially influenced by environmental and human behavioral factors. Those cancers with a higher degree of clustering should be prioritized for the investigation of these risks for further prevention and control strategies. However, which common cancers in China have a relatively higher degree of spatial clustering has yet to be quantified. In this paper, we first compared the spatial clustering degree and high-risk areas of 11 common cancers in mainland China (either separating males and females or pooling them together) and then identified highly clustered cancers requiring special attention to their environmental and behavioral risks, particularly those not yet well studied. We then discussed the potential risk factors resulting in such a high degree of spatial clustering. The findings could offer insights into the spatial epidemiology of cancer and help with targeted monitoring and control.

## Methods

### Data

The cancer data was obtained from “China Cancer Registry Annual Report 2017” (China Cancer Report) edited by the National Cancer Center (NCC) of China [21]. The number of incident cases and crude incidence rates (CIRs) of 11 common cancers with no sex information and cancers by sex were recorded in 339 counties/cities (registries) from January 1, 2014, to December 31, 2014, in China. The quality of cancer registry data was checked by the NCC regarding comparability, completeness, validity and timeliness according to the criteria of the “Guideline for Chinese Cancer Registration 2016” and the criteria of the International Agency for Research on Cancer/International Association of Cancer Registries [21]. The indices used for quality control include the proportion of cases with morphological verification (MV%), mortality to incidence ratio (M/I), and percentage of cases with death certificate only (DCO%). After quality control, 339 registries were included in the published China Cancer Report. These 339 cancer registries are located in 31 provinces (autonomous regions and municipalities) in China, including 129 cities and 210 counties, and covered 288,243,347 people, accounting for 21.07% of the national population in 2014.

Specifically, we investigated esophagus, stomach, liver, colorectum, lung, thyroid, skin, breast, ovarian, prostate and testis cancer. All cancer cases were classified and coded by the International Classification of Diseases version 10 (ICD-10). The population in all registration areas of China for both sexes in 2014 was chosen as the standard population, as its age-specific incidence rates can be obtained from the China Cancer Report. The age-specific population of each registry was calculated by its age-specific population structure obtained from the sixth Chinese national census in 2010 [22] and the number of populations offered by the China Cancer Report. We used the standard population, the number of cases and age-specific population of each registration area and the indirect standardization method to calculate the standardized incidence rates (SIRs) to eliminate the age structure effect [23].

Regarding the environmental data, we used the 2015 product of Global Land Surface Satellite Annual Dynamics of Global Land Cover at 30-m resolution [24]. For each registry, we kept only the built-up areas where people live. We then used the built-up areas to calculate the adjacent distances between the pairs of registries to quantify their spatial relationships. Compared to previous studies using administrative areas [25, 26], our method could improve the accuracy of calculating the spatial weight matrix of the cancer spatial patterns.

### Statistical and spatial analyses

We compared the difference in cancer CIRs and SIRs between males and females using the Poisson regression model. Female incidence rates were used as the reference values when compared with the male incidence rates.

We used the global Moran’s Index (Moran’s I) to quantify the spatial autocorrelation of cancer SIRs in mainland China [27]. The spatial clustering degrees of each cancer across spatial scales were then compared. The Moran’s I was calculated as follows:

1 $$I = \frac{N}{{\sum }_{i=1}^{N}{\sum }_{j=1}^{N}{w}_{i,j}}\frac{{\sum }_{i=1}^{N}{\sum }_{j=1}^{N}{w}_{i,j}\left({x}_{i}-\overline{x }\right)\left({x}_{j}-\overline{x }\right)}{{\sum }_{i=1}^{N}{\left({x}_{i}-\overline{x }\right)}^{2}}$$

where $I$ is the spatial autocorrelation coefficient, $N$ is the number of registries,${x}_{i}$ and ${x}_{j}$ are the standardized incidence rates of the $i$ th and $j$ th registries, $\overline{x }$ is the average standardized incidence rates of cancer for all registries, and ${w}_{i,j}$ is the spatial weight between the $i$ th and $j$ th registries. The value of Moran’s I ranges from -1 to 1. Moran's $I>0$ indicates a clustering tendency, $I=0$ indicates a random tendency, and $I<0$ indicates a dispersion tendency. Meanwhile, the Z-score is used to calculate how statistically significant the clustering tendency is (i.e., the spatial clustering degree). The Z-score was calculated as follows:

2 $${Z}_{I}=\frac{I-{I}_{E}}{\sqrt{Var\left[{I}_{E}\right]}}$$

where ${Z}_{I}$ is the Z-score of $I$, $I$ is the spatial autocorrelation coefficient, ${I}_{E}$ is the expected value of $I$, and $Var\left[{I}_{E}\right]$ is the variance of $I$ for the expected distribution. A smaller p value indicates a higher probability of rejecting the null hypothesis (i.e., the cancer SIR is spatially randomly distributed). When $I>0$, ${Z}_{I}>1.96$($p<0.05$), the clustering is statistically significant [27]. When ${Z}_{I}<1.96$($p\ge 0.05$), the pattern shows no spatial clustering. A higher Z-score indicates a higher spatial clustering degree. Pairwise comparisons between the Z-score were performed using the non-parametric Mann–Whitney U test adjusted by Bonferroni correction. Based on the comparisons, we classified the spatial clustering degree into 4 levels (high, medium, low and no clustering).

The hotspot analysis identifies the locations of local clusters. A statistically significant hotspot is a registry with a high SIR surrounded by other high-SIR registries. The hotspots were identified using the following formulas [28]:

3 $${G}_{i}^{*} = \frac{{\sum }_{j=1}^{N}{w}_{i,j}{x}_{j}- \overline{x}{\sum }_{j=1}^{N}{w}_{i,j} }{S\sqrt{\left[N{\sum }_{j=1}^{N}{w}_{i,j}^{2}- {\left({\sum }_{j=1}^{N}{w}_{i,j}\right)}^{2}\right]/\left(N-1\right)}}$$

4 $$S = \sqrt{\frac{{\sum }_{j=1}^{N}{x}_{j}^{2}}{N}- {\left(\overline{x }\right)}^{2}}$$

where the ${G}_{i}^{*}$ statistic is a Z score, $N$ is the number of registries, ${x}_{j}$ is the standardized incidence rate of the $j$ th registry, $\overline{x }$ is the average value of the standardized incidence rates for all registries, and ${w}_{i,j}$ is the spatial weight between the $i$ th and $j$ th registries. Two significance levels were calculated: ${G}_{i}^{*}>1.96$ ($p<0.05$) means that the hotspot is statistically significant at the 95% confidence interval (CI), and ${G}_{i}^{*}>2.58$ ($p<0.01$) means at the 99% CI. We used the false discovery rate method to adjust the multiple comparisons and spatial dependency effects of the local spatial analysis methods [29].

We used the fixed distance method to calculate the spatial weight matrixes. The adjacent elements within a cut-off distance have equal weights, and the element’s weight beyond this distance is set to zero. The minimum cut-off distance should ensure that each registry has at least one adjacent element after removing distance outliers. The maximum cut-off distance ensures that no element has all other elements as its neighbors. In this study, we set the cut-off distances ranging from 200 to 1,600 km (with an increment of 50 km). The increment is equal to the average nearest neighbor distance. The maximum Z-score indicates the highest degree of spatial clustering. The corresponding cut-off distance was thereby used as the optimal distance to calculate the hotspots. To identify the optimal distance, the global Moran’s I with different cut-off distances were executed. Euclidean distance was used to calculate the adjacent distance between each pair of registries. Finally, the identified cancer hotspot maps were summed to calculate the high-risk areas of the 19 cancers.

The Poisson regression model and non-parametric Mann–Whitney U test were performed in R Statistical software (Version 3.5.1, R Core Team, Vienna, Austria). The global Moran’s I, hotspot analysis and sum of maps were executed in ArcGIS software (Version 10.2, ESRI Inc., Redlands, CA, USA).

## Results

### Summary of cancer incidence

In total, lung cancer had the highest incidence, followed by stomach, liver, colorectum, breast and esophagus cancer in 339 cancer registries (Table 1). Among the cancers by sex, male lung cancer was the most common cancer, followed by female breast, male stomach, male liver, female lung and male colorectum cancer (Table 1). A significant incidence difference between males and females was found in all common cancers except for skin cancer (Table 1). According to the values of the standardized rate ratio (SRR), the risk of esophagus, stomach, liver, lung, and colorectum cancer was higher in males than in females. The risk of female breast and female thyroid cancer was higher than that of males (Table 1).

**Table 1:** Cancer incidence rates and comparison between male and female incidence rates in mainland China, 2014

ICD-10	Cancer	Sex	Freq	CIR (1/10^5^)	SIR (1/10^5^)	Poisson regression		S_Poisson regression	
						**RR**	**95% CI**	**SRR**	**95% CI**
C15	Esophagus	S	58,396		20.26				
		F	16,641	11.72	11.13	1		1	
		M	41,755	28.56	29.76	2.438*	(2.394, 2.482)	2.674*	(2.626, 2.723)
C16	Stomach	S	90,747		31.48				
		F	27,147	19.11	18.31	1		1	
		M	63,600	43.50	45.3	2.276*	(2.244, 2.309)	2.474*	(2.439, 2.510)
C18-21	Colorectum	S	79,180		27.47				
		F	33,623	23.67	22.71	1		1	
		M	45,557	31.16	32.48	1.316*	(1.298, 1.335)	1.430*	(1.410, 1.451)
C22	Liver	S	80,325		27.87				
		F	21,268	14.97	14.36	1		1	
		M	59,057	40.39	41.59	2.698*	(2.656, 2.740)	2.896*	(2.851, 2.943)
C33-34	Lung	S	170,152		59.03				
		F	56,958	40.10	38.35	1		1	
		M	113,194	77.42	80.98	1.931*	(1.911, 1.950)	2.112*	(2.090, 2.133)
C43	Skin	S	1455		0.51				
		F	704	0.50	0.48	1		1	
		M	751	0.51	0.53	1.036	(0.935, 1.149)	1.104	(0.996, 1.224)
C50	Breast	S	60,629		20.75				
		F	59,806	42.11	41.53	1		1	
		M	823	0.56	0.58	0.013*	(0.012, 0.014)	0.014*	(0.013, 0.015)
C56	Ovary	F	10,916	7.69	7.57				
C61	Prostate	M	14,310	9.79	10.55				
C62	Testis	M	656	0.45	0.46				
C73	Thyroid	S	35,435		12.29				
		F	26,589	18.72	18.59	1		1	
		M	8846	6.05	6.1	0.323*	(0.316, 0.331)	0.328*	(0.320, 0.336)

*Freq* Frequency, *CIR* Crude incidence rate, *SIR* Standardized incidence rate, *S_Poisson regression* poisson regression was performed using SIR, *RR* Rate ratio, *SRR* Standardized rate ratio, CI Confidence interval, *S* Sum of males and females, *F* Female, *M* Male, *Colorectum* Colon, rectum and anus, *Lung* Trachea, bronchus and lung, *Skin* Melanoma of skin, *: *p* < 0.05

### Spatial clustering degree

**Fig. 2:**
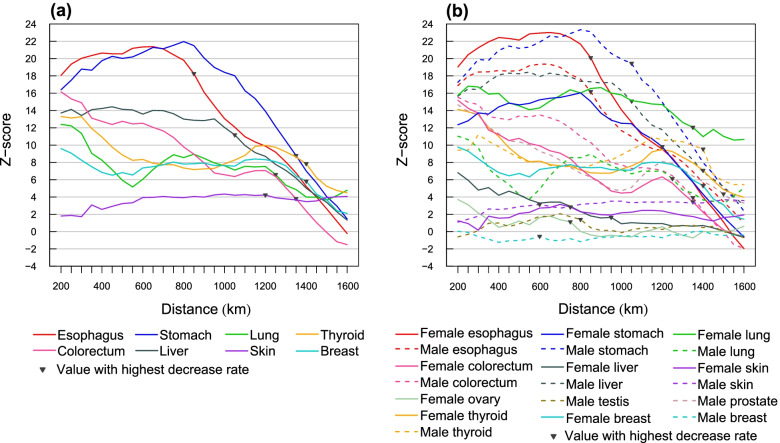
Z-score of global Moran’s Index for the cancer standardized incidence rates of (**a**) cancers with no sex information and (**b**) cancers by sex in mainland China in 2014. Colorectum: colon, rectum and anus; Lung: trachea, bronchus and lung; Skin: melanoma of skin

**Fig. 3:**
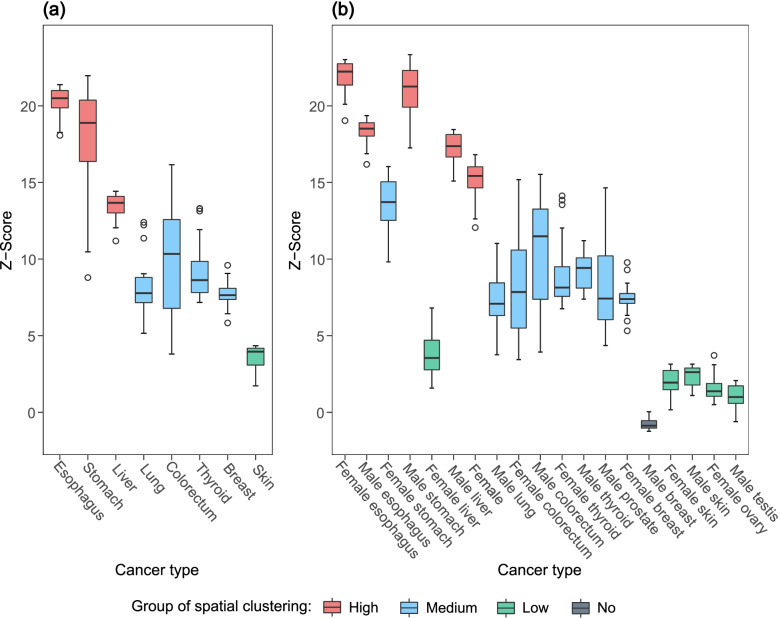
Spatial clustering degree of (**a**) cancers with no sex information and (**b**) cancers by sex in mainland China in 2014. Colorectum: colon, rectum and anus; Lung: trachea, bronchus and lung; Skin: melanoma of skin. Four groups were formed based on the levels of difference in Z-Score tested by the non-parametric Mann–Whitney U test (Tables S1 and S2 in Additional file 1)

The spatial clustering degree was compared using the medians of Z-scores ranging from the smallest distance (200 km) to the distance where the highest rate of value decrease occurs (marked as triangles in Fig. 2). The results of pairwise comparisons between cancers by the non-parametric Mann–Whitney U test are shown in Tables S1 and S2 (Additional file 1). Esophagus, stomach and liver cancer had a significantly higher degree of spatial clustering ($p<0.01$, Fig. 3a). When splitting males and females, female esophagus, male stomach, male esophagus, male liver and female lung cancer showed significantly higher clustering degree ($p<0.001$, Fig. 3b). The spatial clustering degree in male liver cancer was significantly higher than that in female liver cancer ($p<0.001$). The spatial clustering degree of female lung cancer was significantly higher than that of male lung cancer ($p<0.001$). Lung, colorectum, thyroid, breast, female stomach, male colorectum, male thyroid, female thyroid, female colorectum, prostate, female breast, and male lung cancer had medium clustering degree. The spatial clustering degree in skin, male skin, female skin, female liver, female ovarian and male testis cancer was relatively low. A significant spatial clustering was found in all cancers except for male breast cancer. Figure S1 (Additional file 1) shows the values of the Moran’s I. Table S3 (Additional file 1) shows the Moran’s I and Z-score at the distance where the highest degree of clustering occurs.

### Cancer hot spots

**Fig. 4:**
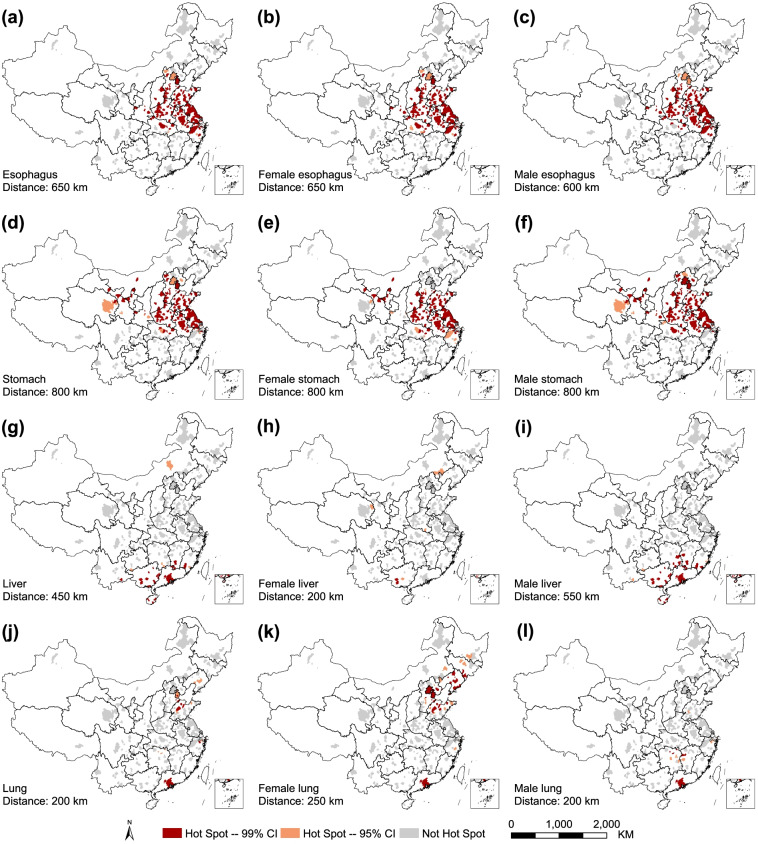
Hotspots for cancers with a high degree of spatial clustering in mainland China, 2014. For comparison, e, h, j, and l (with medium or low degree of clustering) are included. (**a**) esophagus; (**b**) female esophagus; (**c**) male esophagus; (**d**) stomach; (**e**) female stomach; (**f**) male stomach; (**g**) liver; (**h**) female liver; (**i**) male liver; (**j**) lung (trachea, bronchus and lung); (**k**) female lung; (**l**) male lung

The statistically significant hotspots ($p<0.05$) in 339 cancer registries were found in all cancers except for male breast, male skin, female skin and male testis under the optimal distance. Esophagus cancer was mainly found in North China, the Huai River Basin, the Yangtze River Delta region and Shaanxi Province (Fig. 4a-c). The spatial patterns of hotspots in stomach cancer were similar to those in the esophagus but were also found in western Inner Mongolia, Ningxia, Gansu and Qinghai Province (Fig. 4d-f). The hotspots for liver and male liver cancer were distributed in Southeast China (Guangxi, Pearl River Delta region, Hainan, south Jiangxi and south Fujian) and south Hunan Province (Fig. 4g, i). High-risk areas of female liver cancer were found in Guangxi, Qinghai and Inner Mongolia (Fig. 4h). Except for in the Pearl River Delta region, the spatial distribution of hotspots in male lung cancer was very different from that in female lung cancer (Fig. 4j-l). Hotspots of female lung cancer were mainly located in Shandong, North and Northeast China, whereas hotspots for male lung cancer were located in Hunan and Jiangxi Province. The hotspots of colorectum, male colorectum and female colorectum cancer were located in the Yangtze River Delta and Pearl River Delta (Figure S2a-S2c, Additional file 1). Extra hotspots for colorectum and male colorectum were also found in Shandong Peninsula, Liaoning and Fujian. The hotspots of thyroid cancer were found in the Yangtze River Delta, and extra hotspots for male thyroid cancer were found in Hebei and Xinjiang (Figure S2d-S2f, ﻿Additional file 1). Hotspots for breast and female breast cancer were found in Shandong, Hebei, Tianjin, Beijing, northern Zhejiang, Liaoning and the Pearl River Delta (Figure S2g, S2h, ﻿Additional file 1). The high-risk clusters of prostate cancer were located in the Yangtze River Delta and Pearl River Delta (Figure S2i, ﻿Additional file 1). The hotspots of skin cancer were distributed in Guangxi, Jiangxi, Fujian, Zhejiang and Hunan (Figure S2j, ﻿Additional file 1). A high-risk area of ovarian cancer was found in the Pearl River Delta region (Figure S2k, ﻿Additional file 1). Moreover, the Yangtze River Delta and Pearl River Delta were the hotspots for at least 7 types of cancers by sex (Figure S3, Additional file 1).

## Discussion

This study compared the spatial clustering degree and high-risk areas of 11 common cancers and cancers by sex in mainland China, identified the knowledge gap in understanding the spatial pattern of cancer burdens and discussed potential risk factors at work. Esophagus, stomach and liver cancer had a significantly higher degree of spatial clustering ($p<0.01$). For cancers by sex, female esophagus, male stomach, male esophagus, male liver and female lung cancer were the top 5 cancers with a significantly higher degree of spatial clustering ($p<0.001$). The spatial clustering degree in male liver cancer was significantly higher than that of female liver cancer and so as female lung cancer in comparison with male lung cancer ($p<0.001$). Cancers with a high spatial clustering degree indicate that potential environmental and behavioral factors, such as unhealthy diets, water pollution and climate factors are at work. Further understanding of the potential environmental and behavioral factors driving the strong clustering is needed, particularly for male liver cancer, which has rarely been investigated based on our literature review. The identified hotspots could be used as representative areas for future studies and targets for cancer monitoring and intervention.

The spatial clustering degree for male liver cancer was high, but knowledge of its spatial pattern and potential environmental and behavioral drivers was limited. One possible reason could be that the incidence rate of male liver cancer has slowly decreased in the last two decades [3]. In contrast, fast-increasing cancers such as female breast and female lung cancer have received more attention.

The high spatial clustering degree of esophagus and stomach (except for female stomach) cancer might be due to their correlation with the spatial patterns of unhealthy diets, drinking water pollution [26] and specific climate conditions [30]. For example, 30% of esophagus deaths and cases were attributable to low intake of fruits and vegetables in China [31], and pickled food (OR = 1.59, $p<0.01$) and low intake of fruit (OR = 1.49, $p<0.01$) were strongly associated with the development of stomach cancer in Jiangsu [32]. The consumption of pickled food is common [26] in esophagus cancer high-risk areas, such as North China and the Huai River Basin in China. Unhealthy diets, such as pickled food and low intake of fruits and vegetables [33, 34], might be associated with the high stomach cancer risk in western Inner Mongolia, Ningxia and Gansu. Alcohol drinking is also an important risk factor for esophagus and stomach cancer [35], and the provinces with the high prevalence of alcohol use in males, such as Shandong, Henan and Jiangsu [36], were also high-risk areas in male esophagus and male stomach. Drinking water pollution (e.g., organic matter, heavy metals and nitrate/nitrite) is one of the leading environmental risk factors for stomach and esophagus cancer [9, 37]. Highly polluted river basins are mainly located in the Huai, Hai, Yellow, and Liao River Basins [38], which match well with the identified high-risk areas of esophagus and stomach cancer. In addition, climate factors such as low rainfall and drought can facilitate the formation of nitrosamines and precursors [39], a threat to digestive tract cancers in Northwest China.

Male liver cancer showed a distinct degree of spatial clustering with female liver cancer. A possible explanation is that males are more likely to drink alcohol [40] and be affected by the hepatitis virus [41]. Shen et al. found that alcohol consumption and hepatitis B virus (HBV) mortality were positively associated with male liver mortality but not significantly associated with female liver mortality [40]. The burdens of HBV and hepatitis C virus (HCV) were also much higher in males than in females [41], and high-risk clusters on the incidence of HBV and HCV were found in areas of Southeast China [42]. Moreover, low economic level [43], unhealthy dietaries [44], water pollution [45] and other climate factors such as rain, heat and humidity, which facilitate the aflatoxin contamination in food (e.g., maize and peanuts) [46]. This might be the reason for the high spatial clustering of liver cancer. For example, we found that some high-risk clusters of liver cancer were located in underdeveloped central and western regions, such as Guangxi and Hunan. Exposure to arsenic can induce liver cancer [47], and a study found that the spatial distribution of arsenic water pollution showed significant spatial clustering [48]. People living in the Pearl River Delta, Guangxi and Fujian might prefer the diet of raw seafood, leading to the high prevalence of liver fluke infection [49], which caused liver fibrosis and cirrhosis and thereby increased the risk of liver cancer [50].

The female lung cancer pattern showed a significantly higher degree of spatial clustering than male lung cancer. These difference might be caused by the spatial distribution of male smokers not being clustered but being more spatially concentrated in females [51]. One possible reason for the high-risk areas of female lung cancer in Northeast China and Tianjin is that the prevalence of both active smoking and second-hand smoking in females is higher [52]. Another reason might be that non-smoking women are easy to be exposed to indoor air pollution produced by cooking and coal burning in the prolonged winter in the north [53]. Air pollution facilitates the development of lung cancer [54, 55] and is heavily spatial clustered. Heavy pollution was found in North China, the Yangtze River Delta, the Huai River Basin [56–59], and the Pearl River Delta [60], which matches with our identified high-risk lung cancer clusters.

We observed both similarities and differences between the identified cancer hotspots in 2014 and traditional high-risk areas in China. Cixian and Shexian in Hebei Province and Linxian in Henan Province, which are located in the transition zone between the Taihang Mountains and North China Plain, were defined as typical high-risk areas of Chinese esophagus cancer since the 1970s [61]. The incidence and mortality of esophagus cancer in these areas have decreased after long-term prevention and control but are still much higher than other regions. We did not find high-risk clustering in Qidong, a typical area with high incidence of liver cancer since the 1970s [44]. This could be explained by effective intervention, prevention and screening measures, such as improving drinking water environment and HBV vaccination in the past 40 years [62].

We found weak or no spatial clustering in melanoma of skin, male breast and testicular cancer. Geographic variation in skin cancer has been found in southwestern Sweden [63] and Iran [64]. However, melanoma of skin has very low incidence rates in China, probably due to much less exposure to sunlight [65]. Male breast cancer is a rare carcinoma, and there is no evidence of its spatial clustering [66]. A study using population-level data in Britain [67] and a study using individual-level data in Denmark [68] did not find evidence of spatial clustering in testicular cancer. Environmental or human behavioral factors might not play a strong role in the spatial pattern of these cancers.

The Yangtze River Delta and the Pearl River Delta were high-risk areas for multiple cancers, which might be due to the spatial aggregation of a high socioeconomic status (SES), high-fat diets, and heavier air pollution. At first, a higher SES is associated with the increasing risk of cancers, such as thyroid and female breast cancer [69, 70]. Higher SES could increase cancer incidence detection through widespread application of screening techniques [71]. For female breast cancer, another mechanism is that high SES increases the risk by a higher access to hormone replacement therapy, oral contraceptive, and later age at first birth [72, 73]. In contrast, a case–control study in South Carolina, USA, found that a high educational level representing high SES was associated with a lower risk of prostate cancer [74], which might be due to healthy lifestyles such as less drinking and smoking. Secondly, the transition in diets from high fiber and carbohydrate to high fat that is related to the improvement of the economy [75] can increase the prevalence of obesity and overweight, which are important risk factors for colorectum, breast and prostate cancer. Finally, the concentration of PM2.5 in these areas is much higher than the annual average World Health Organization Air Quality Guideline [76–78]. Positive associations have been reported between air pollution and the risk of lung, colorectum, stomach and liver cancer [11, 79–81]. A cohort study in Taiwan, China, also found that long-term exposure to PM2.5 could increase the mortality of liver cancer and colorectal cancer [82].

There are some limitations of the study. First, the address of the cancer case obtained by the household registration system might not represent the actual place of residence, which might affect the accuracy of the interpretations about the effects of environmental factors on cancers. However, cancer is most frequently diagnosed in elderly population, and the separation of registered and actual residences among the elderly in China is rare, so the locations of the registration addresses could reflect their living conditions. Nevertheless, we recommend that the NCC provide the cancer data of the resident population, in addition to the household registration population, to help improve the accuracy. When the individual address of the cancer case is available, it could be used to further improve the accuracy. Second, the number of cancer registries in western areas is much smaller than that in other areas due to disparities in public health resources and economic development levels. The identified spatial patterns might be affected by the distribution of cancer registries, e.g., the cancer burden in western areas, which could be underestimated [26]. The clustering patterns might change when the number of cancer registries increases, our findings could be further validated using the up-to-date cancer data. The government needs to increase the number of cancer registries in western areas and improve the fairness of the distribution of public health resources. This would further improve the accuracy of quantifying cancer spatial patterns and potential environmental and behavioral risks.

## Conclusions

Esophagus, stomach and liver cancer showed a significantly higher degree of spatial clustering than other cancers. Female esophagus, male stomach, male esophagus, male liver and female lung cancer were among the top 5 highly clustered cancer types by sex. The spatial clustering degree of liver cancer was significantly higher in males than in females, as was that of lung cancer in females in comparison to males. Among the top highly clustered cancers, knowledge of their spatial patterns and environmental and behavioral risk factors is generally limited. Potential factors, such as unhealthy diets, water pollution and climate factors have been suggested, and further investigation and validation are urgently needed, particularly for male liver cancer. Moreover, shared environmental or behavioral risks should be further studied for effective control of risk areas of multiple cancers.

## Supplementary Information

**Additional file 1: Figure S1.** Moran’s I of global Moran’s Index for the cancer standardized incidence rates of (a) cancers with no sex information and (b) cancers by sex in mainland China in 2014. Colorectum: colon, rectum and anus; Lung: trachea, bronchus and lung; Skin: melanoma of skin. **Table S1.** The *p*-value of pairwise comparison between cancers with no sex information by the Mann–Whitney U test (*p* < 0.05). **Table S2.** The *p*-value of pairwise comparison between cancers by sex information by the non-parametric Mann–Whitney U test (*p* < 0.05). **Table S3.** Moran’s I and Z-score of cancers (only the ones with significant effects are shown) in mainland China in 2014 and the distance where the highest degree of clustering occurs. **Figure S2 **Hotspots for cancers with relatively medium and low degree of spatial clustering in mainland China, 2014. Medium degree of clustering: (a) colorectum (colon, rectum and anus); (b) female colorectum; (c) male colorectum; (d) thyroid; (e) female thyroid; (f) male thyroid; (g) breast; (h) female breast; (i) male prostate. Low degree of clustering: (j) skin (melanoma of skin); (k) female ovary. **Figure S3. **Numbers of cancer types by sex that have overlapped hotspot areas in mainland China, 2014.

## Data Availability

All data generated or analyzed during this study are included in this published article.

## Abbreviations

Lung: Trachea, bronchus, and lung
Colorectum: Colon, rectum, and anus
PM: Particulate matter
Skin: Melanoma of skin
NCC: National Cancer Center
CIRs: Crude incidence rates
MV%: Proportion of cases with morphological verification
M/I: Mortality to incidence ratio
DCO%: Percentage of cases with death certificate only
ICD-10: International Classification of Diseases version 10
SIRs: Standardized incidence rates
Moran’s I: Moran’s Index
CI: Confidence interval
SRR: Standardized rate ratio
HBV: Hepatitis B virus
HCV: Hepatitis C virus
SES: Socioeconomic status
