# Supplementary material for: Comparing spatial patterns of 11 common cancers in Mainland China
Source: BMC Public Health. 2022 Aug 15;22:1551. doi: 10.1186/s12889-022-13926-y (PMC9377081; doi:10.1186/s12889-022-13926-y)
Supplement: Supplementary file 1 — Additional file 1: Figure S1. Moran’s I of global Moran’s Index for the cancer standardized incidence rates of (a) cancers with no sex information and (b) cancers by sex in mainland China in 2014. Colorectum: colon, rectum and anus; Lung: trachea, bronchus and lung; Skin: melanoma of skin. Table S1. The p-value of pairwise comparison between cancers with no sex information by the Mann–Whitney U test (p < 0.05). Table S2. The p-value of pairwise comparison between cancers by sex information by the non-parametric Mann–Whitney U test (p < 0.05). Table S3. Moran’s I and Z-score of cancers (only the ones with significant effects are shown) in mainland China in 2014 and the distance where the highest degree of clustering occurs. Figure S2 Hotspots for cancers with relatively medium and low degree of spatial clustering in mainland China, 2014. Medium degree of clustering: (a) colorectum (colon, rectum and anus); (b) female colorectum; (c) male colorectum; (d) thyroid; (e) female thyroid; (f) male thyroid; (g) breast; (h) female breast; (i) male prostate. Low degree of clustering: (j) skin (melanoma of skin); (k) female ovary. Figure S3. Numbers of cancer types by sex that have overlapped hotspot areas in mainland China, 2014. [file 12889_2022_13926_MOESM1_ESM.docx]

**Figure S1** Moran’s I of global Moran’s Index for the cancer standardized incidence rates of (a) cancers with no sex information and (b) cancers by sex in mainland China in 2014. Colorectum: colon, rectum and anus; Lung: trachea, bronchus and lung; Skin: melanoma of skin.

**Table S1** The p-value of pairwise comparison between cancers with no sex information by the Mann–Whitney U test (p < 0.05).

| **Cancers** | **Esophagus** | **Stomach** | **Liver** | **Lung** | **Colorectum** | **Thyroid** | **Breast** | **Skin** |
| --- | --- | --- | --- | --- | --- | --- | --- | --- |
| Esophagus | 1.000 |  |  |  |  |  |  |  |
| Stomach | 1.000 | 1.000 |  |  |  |  |  |  |
| Liver | <0.001 | <0.001 | 1.000 |  |  |  |  |  |
| Lung | <0.001 | <0.001 | <0.001 | 1.000 |  |  |  |  |
| Colorectum | <0.001 | <0.001 | 0.004 | 1.000 | 1.000 |  |  |  |
| Thyroid | <0.001 | <0.001 | <0.001 | 1.000 | 1.000 | 1.000 |  |  |
| Breast | <0.001 | <0.001 | <0.001 | 1.000 | 1.000 | 0.027 | 1.000 |  |
| Skin | <0.001 | <0.001 | <0.001 | <0.001 | <0.001 | <0.001 | <0.001 | 1.000 |

Colorectum: colon, rectum and anus; Lung: trachea, bronchus and lung; Skin: melanoma of skin

**Table S2** The p-value of pairwise comparison between cancers by sex information by the non-parametric Mann–Whitney U test (p < 0.05).

| **Cancers** | **F**  **Esopha**  **-gus** | **M**  **Esopha**  **-gus** | **F Stomach** | **M Stomach** | **F**  **Liver** | **M**  **Liver** | **F**  **Lung** | **M**  **Lung** | **F**  **Colorec**  **-tum** | **M**  **Colorec**  **-tum** | **F**  **Thyroid** | **M Thyroid** | **M Prostate** | **F**  **Breast** | **M**  **Breast** | **F**  **Skin** | **M**  **Skin** | **F**  **Ovary** | **M**  **Testis** |
| --- | --- | --- | --- | --- | --- | --- | --- | --- | --- | --- | --- | --- | --- | --- | --- | --- | --- | --- | --- |
| F Esophagus | 1.000 |  |  |  |  |  |  |  |  |  |  |  |  |  |  |  |  |  |  |
| M Esophagus | <0.001 | 1.000 |  |  |  |  |  |  |  |  |  |  |  |  |  |  |  |  |  |
| F Stomach | <0.001 | 0.000 | 1.000 |  |  |  |  |  |  |  |  |  |  |  |  |  |  |  |  |
| M Stomach | 1.000 | 0.001 | <0.001 | 1.000 |  |  |  |  |  |  |  |  |  |  |  |  |  |  |  |
| F Liver | <0.001 | <0.001 | <0.001 | <0.001 | 1.000 |  |  |  |  |  |  |  |  |  |  |  |  |  |  |
| M Liver | <0.001 | 0.403 | <0.001 | <0.001 | <0.001 | 1.000 |  |  |  |  |  |  |  |  |  |  |  |  |  |
| F Lung | <0.001 | <0.001 | 0.411 | <0.001 | <0.001 | <0.001 | 1.000 |  |  |  |  |  |  |  |  |  |  |  |  |
| M Lung | <0.001 | <0.001 | <0.001 | <0.001 | <0.001 | <0.001 | <0.001 | 1.000 |  |  |  |  |  |  |  |  |  |  |  |
| F Colorectum | <0.001 | <0.001 | <0.001 | <0.001 | <0.001 | <0.001 | <0.001 | 1.000 | 1.000 |  |  |  |  |  |  |  |  |  |  |
| M Colorectum | <0.001 | <0.001 | 0.247 | <0.001 | <0.001 | <0.001 | <0.001 | 0.111 | 1.000 | 1.000 |  |  |  |  |  |  |  |  |  |
| F Thyroid | <0.001 | <0.001 | <0.001 | <0.001 | <0.001 | <0.001 | <0.001 | 1.000 | 1.000 | 1.000 | 1.000 |  |  |  |  |  |  |  |  |
| M Thyroid | <0.001 | <0.001 | <0.001 | <0.001 | <0.001 | <0.001 | <0.001 | 0.040 | 1.000 | 1.000 | 1.000 | 1.000 |  |  |  |  |  |  |  |
| M Prostate | <0.001 | <0.001 | <0.001 | <0.001 | <0.001 | <0.001 | <0.001 | 1.000 | 1.000 | 1.000 | 1.000 | 1.000 | 1.000 |  |  |  |  |  |  |
| F Breast | <0.001 | <0.001 | <0.001 | <0.001 | <0.001 | <0.001 | <0.001 | 1.000 | 1.000 | 0.290 | 0.679 | <0.001 | 1.000 | 1.000 |  |  |  |  |  |
| M Breast | <0.001 | <0.001 | <0.001 | <0.001 | <0.001 | <0.001 | <0.001 | <0.001 | <0.001 | <0.001 | <0.001 | <0.001 | <0.001 | <0.001 | 1.000 |  |  |  |  |
| F Skin | <0.001 | <0.001 | <0.001 | <0.001 | 0.114 | <0.001 | <0.001 | <0.001 | <0.001 | <0.001 | <0.001 | <0.001 | <0.001 | <0.001 | 0.001 | 1.000 |  |  |  |
| M Skin | <0.001 | <0.001 | <0.001 | <0.001 | 1.000 | <0.001 | <0.001 | <0.001 | <0.001 | <0.001 | <0.001 | <0.001 | <0.001 | <0.001 | 0.007 | 1.000 | 1.000 |  |  |
| F Ovary | <0.001 | <0.001 | <0.001 | <0.001 | 0.029 | <0.001 | <0.001 | <0.001 | <0.001 | <0.001 | <0.001 | <0.001 | <0.001 | <0.001 | 0.001 | 1.000 | 1.000 | 1.000 |  |
| M Testis | <0.001 | <0.001 | <0.001 | <0.001 | <0.001 | <0.001 | <0.001 | <0.001 | <0.001 | <0.001 | <0.001 | <0.001 | <0.001 | <0.001 | 0.021 | 1.000 | 0.132 | 1.000 | 1.000 |

F: female; M: male; Colorectum: colon, rectum and anus; Lung: trachea, bronchus and lung; Skin: melanoma of skin

**Table S3** Moran’s I and Z-score of cancers (only the ones with significant effects are shown) in mainland China in 2014 and the distance where the highest degree of clustering occurs.

| **Cancers** | **Sex** | **The optimal distance (km)** | **Moran’s I** | **Z-score** |
| --- | --- | --- | --- | --- |
| Esophagus | S | 650 | 0.22 | 21.38 |
|  | F | 650 | 0.24 | 23.02 |
|  | M | 600 | 0.22 | 19.36 |
| Stomach | S | 800 | 0.18 | 21.97 |
|  | F | 800 | 0.13 | 16.04 |
|  | M | 800 | 0.19 | 23.34 |
| Liver | S | 450 | 0.2 | 14.43 |
|  | F | 200 | 0.18 | 6.81 |
|  | M | 550 | 0.22 | 18.45 |
| Colorectum | S | 200 | 0.43 | 16.16 |
|  | F | 200 | 0.4 | 15.18 |
|  | M | 200 | 0.42 | 15.53 |
| Lung | S | 200 | 0.33 | 12.4 |
|  | F | 250 | 0.38 | 16.81 |
|  | M | 200 | 0.3 | 11.02 |
| Thyroid | S | 200 | 0.35 | 13.31 |
|  | F | 200 | 0.37 | 14.13 |
|  | M | 300 | 0.22 | 11.2 |
| Breast | S | 200 | 0.26 | 9.59 |
|  | F | 200 | 0.26 | 9.78 |
| Skin | S | 1000 | 0.03 | 4.34 |
|  | F | 700 | 0.03 | 3.15 |
|  | M | 1500 | 0.01 | 3.68 |
| Ovary | F | 200 | 0.1 | 3.71 |
| Prostate | M | 200 | 0.39 | 14.65 |
| Testis | M | 700 | 0.02 | 2.07 |

S: sum of male and female; F: female; M: male; Colorectum: colon, rectum and anus; Lung: trachea, bronchus and lung; Skin: melanoma of skin





**Figure S2** Hotspots for cancers with relatively medium and low degree of spatial clustering in mainland China, 2014. Medium degree of clustering: (a) colorectum (colon, rectum and anus); (b) female colorectum; (c) male colorectum; (d) thyroid; (e) female thyroid; (f) male thyroid; (g) breast; (h) female breast; (i) male prostate. Low degree of clustering: (j) skin (melanoma of skin); (k) female ovary.


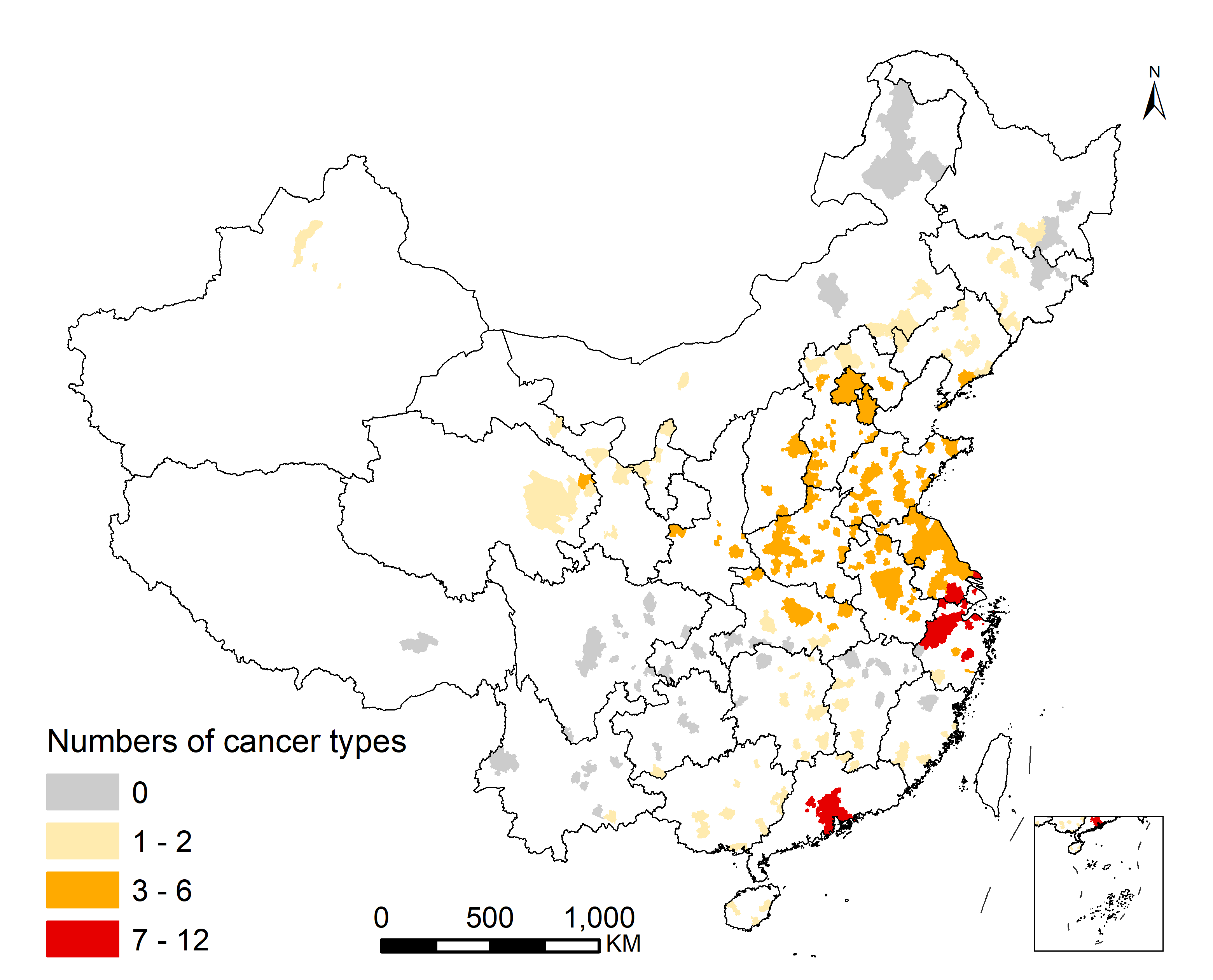


**Figure S3** Numbers of cancer types by sex that have overlapped hotspot areas in mainland China, 2014
